# Prevalence of Prediabetes and Diabetes Mellitus Type II in Bipolar Disorder

**DOI:** 10.3389/fpsyt.2020.00314

**Published:** 2020-04-22

**Authors:** Sarah Kittel-Schneider, Daniel Bury, Karolina Leopold, Sara Haack, Michael Bauer, Steffi Pfeiffer, Cathrin Sauer, Andrea Pfennig, Henry Völzke, Hans-Jörgen Grabe, Andreas Reif

**Affiliations:** ^1^ Department of Psychiatry, Psychosomatic Medicine and Psychotherapy, University Hospital, Goethe University, Frankfurt, Germany; ^2^ Department of Psychiatry, Psychosomatic Medicine and Psychotherapy, University Hospital, Julius-Maximilians-University of Würzburg, Würzburg, Germany; ^3^ Department of Psychiatry and Psychotherapy Munich East, kbo-Isar-Amper-Klinikum, Haar, Germany; ^4^ Department of Psychiatry and Psychotherapy, Carl Gustav Carus University Hospital, Medical Faculty, Technische Universität Dresden, Dresden, Germany; ^5^ Department of Psychiatry, Psychotherapy and Psychosomatics, Vivantes Hospital am Urban and Vivantes Hospital im Friedrichshain, Charite Universitätsmedizin, Berlin, Germany; ^6^ Institute for Community Medicine, University Medicine Greifswald, Greifswald, Germany; ^7^ Department of Psychiatry and Psychotherapy, University Medicine Greifswald, Greifswald, Germany

**Keywords:** bipolar disorder, diabetes mellitus, prediabetes, affective disorders, metabolic syndrome, glucose metabolism, obesity, body mass index

## Abstract

**Introduction:**

Bipolar disorder (BD) is characterized by recurrent episodes of depression and mania and affects up to 2% of the population worldwide. Patients suffering from bipolar disorder have a reduced life expectancy of up to 10 years. The increased mortality might be due to a higher rate of somatic diseases, especially cardiovascular diseases. There is however also evidence for an increased rate of diabetes mellitus in BD, but the reported prevalence rates vary by large.

**Material and Methods:**

85 bipolar disorder patients were recruited in the framework of the BiDi study (Prevalence and clinical features of patients with Bipolar Disorder at High Risk for Type 2 Diabetes (T2D), at prediabetic state and with manifest T2D) in Dresden and Würzburg. T2D and prediabetes were diagnosed measuring HBA1c and an oral glucose tolerance test (oGTT), which at present is the gold standard in diagnosing T2D. The BD sample was compared to an age-, sex- and BMI-matched control population (n = 850) from the Study of Health in Pomerania cohort (SHIP Trend Cohort).

**Results:**

Patients suffering from BD had a T2D prevalence of 7%, which was not significantly different from the control group (6%). Fasting glucose and impaired glucose tolerance were, contrary to our hypothesis, more often pathological in controls than in BD patients. Nondiabetic and diabetic bipolar patients significantly differed in age, BMI, number of depressive episodes, and disease duration.

**Discussion:**

When controlled for BMI, in our study there was no significantly increased rate of T2D in BD. We thus suggest that overweight and obesity might be mediating the association between BD and diabetes. Underlying causes could be shared risk genes, medication effects, and lifestyle factors associated with depressive episodes. As the latter two can be modified, attention should be paid to weight changes in BD by monitoring and taking adequate measures to prevent the alarming loss of life years in BD patients.

## Introduction 

Bipolar disorder (BD) is characterized by recurrent episodes of depression and mania and affects up to 2% of the population worldwide. Patients suffering from BD have a reduced life expectancy of 10 years. The increased mortality, besides mortality caused by suicides, might be due to a higher rate of somatic diseases, especially cardiovascular diseases (1–7). There is also evidence that bipolar patients might have a higher risk for developing diabetes mellitus type II (T2D) (8). However, it remains unclear if the higher rates of somatic diseases and especially diabetes mellitus are caused by psychotropic medication, an unhealthier lifestyle, genetic risk factors, inflammatory mechanisms or shared pathophysiological mechanisms, or a combination of those factors. Additionally, the reported prevalence rates of T2D in BD vary from 6.7 to 26% in different populations (9–12).

Results from epidemiological studies estimate that the risk for T2D in bipolar patients is about threefold increased in comparison to nonpsychiatric populations (13). Conversely, in cohorts of diabetic patients higher comorbidity with psychiatric and especially affective disorders can be found (14). One factor conveying the risk of T2D in BD might be psychotropic medication, especially second generation antipsychotics (15–17). But also lithium and valproic acid are known to induce weight gain and by this could lead to dysregulation in glucose metabolism (18, 19).

However, dysregulation in glucose metabolism in BD patients has been described before the use of second generation antipsychotic medication as well as in drug-naïve patients (20). Therefore, other factors might also play a role, such as shared heritability due to shared risk gene variants. However, in a Japanese sample no association of risk genes of T2D with BD could be detected (21, 22) which was later confirmed in the largest GWAS to date. Notably however, there was a nominally significant correlation of bipolar disorder with body mass index, and in pathway analyses, genes involved in insulin secretion were enriched (23). A recent cohort study investigating 10,863 Danish men reported also an increased rate of T2D in patients with severe mental illness, which however was more pronounced in schizophrenia patients (HR = 1.92; 95%CI, 1.61–2.30). A Swedish study found a much stronger risk increase of cardiovascular disease in patients with schizophrenia and BD as compared to T2D risk (24). In an Amish family study, a positive genetic correlation of BD and T2D was found; however, this was a very distinct population so it is not clear whether this holds true to broader population samples (25).

Regarding environmental risk contributing to T2D in BD, several studies report an unhealthy life style in patients including physical inactivity especially in depressive phases (26, 27), higher alcohol and illegal substance consumption, nicotine dependence and greater intake of unhealthy food (28–31), and increased rates of psychological trauma/maltreatment in childhood (32, 33). Additionally, several endocrine and metabolic pathways could be playing a role in conveying a greater risk of T2D in BD, such as dysregulation of different neuropeptides (for example leptin, ghrelin, and adiponectin) and disturbances in the hypothalamus–pituitary–adrenal gland axis (34, 35). Furthermore, inflammatory and immune processes have been suggested to play a pathophysiological role in T2D as well as BD (34). In an own previous study, we could find hints for an increased prevalence of T2D in bipolar patients; however, we did not include a BMI-, sex- and age-matched control population (36). As there are inconsistent results regarding the prevalence of T2D and BD and the causal mechanisms are still unclear, we here investigated the prevalence of T2D and prediabetes in BD patients in comparison to an age-, sex- and BMI-matched control sample from the general population (37).

## Material and Methods

### Participants

#### Patients

Bipolar disorder patients were recruited in the framework of the BiDi study (Prevalence and clinical features of patients with Bipolar Disorder at High Risk for Type 2 Diabetes (T2D), at prediabetic state and with manifest T2D). This study was a cross-sectional study which was conducted as a collaborative study of the Department of Psychiatry and Psychosomatic Medicine of the University Hospital of Dresden and the Department of Psychiatry, Psychosomatic Medicine and Psychotherapy of the University Hospital of Würzburg. Patients were recruited from the specialized bipolar clinics in Dresden and Würzburg between November 2009 and February 2012 and were mainly outpatients. All participants were diagnosed with a bipolar disorder using ICD-10 criteria from two independent specialists (SKS/AR and SH/KL). Inclusion criteria were age >= 18 years, being euthymic for 2 months [measured by Montgomery–Åsberg Depression Rating Scale (MADRS), Young Mania Rating Scale (YMRS), and Clinical Global Impression Scale Bipolar Disorder (CGI-BP S)]. Only euthymic patients were included, which was operationalized as a score ≤ 12 in the MADRS, ≤5 in the YMRS, and ≤2 in the CGI-BP Score. Medication had to be stable for at least 2 months. Exclusion criteria were organic affective disorder, acute or severe medical conditions (like acute and chronic infections, digestive diseases, carcinomas), pregnant and lactating women.

Diabetes mellitus type II (T2D) was diagnosed using the criteria of the *American Diabetes Association* (ADA) (38) for T2D which are:

- HbA1c ≥ 6.5%
- or fasting glucose ≥ 126 mg/dl (7.0 mmol/L)
- or 2 h plasma glucose ≥ 200 mg/dl (11.1 mmol/L) in the oral glucose tolerance test (oGTT)
- or glucose level at a random time point ≥ 200 mg/dl (11.1 mmol/l) and other symptoms of a diabetes mellitus

Prediabetes was also diagnosed following the criteria of the *American Diabetes Association* (ADA) (38) which are:

- Impaired fasting glucose (IFG): 100–125 mg/dl (5.6–6.9 mmol/L)
- Impaired 2 h plasma glucose (IGT) in the oGTT: 140–199 mg/dl (7.8–11.0 mmol/L)
- HbA1c values between 5.7 and 6. 4%

Only study participants who gave written informed consent were enrolled in the study, which complied with the latest Declaration of Helsinki and was approved by the Ethics Committees of the Universities of Würzburg and Dresden.

#### Healthy Comparison Group: Study of Health in Pomerania

As a mentally healthy comparison group, data from the Study of Health in Pomerania (SHIP study) were used (37, 39, 40). SHIP is a general population cohort study in Northeastern Germany that includes two independent cohorts. The baseline assessment of the first cohort (SHIP-0) was conducted between 1997 and 2001; 4,308 adults were included. Follow-up assessments were conducted between 2002 and 2006 (SHIP-1) and from 2007 to 2012 (SHIP-2). In parallel to the SHIP-2 recruitments, a second, independent cohort was selected in 2008 with 8,016 adults (SHIP-Trend). From this cohort, 4,420 adults were recruited for the basic assessment between 2008 and 2012. Inclusion criteria were age between 20 and 79, German nationality, and living in the Northeastern region. SHIP is an epidemiological study and had the primary aim to investigate the prevalence and incidence of population relevant diseases and risk factors for those diseases. By the comparison of two cross-sectional studies, (SHIP-0 and SHIP-Trend), prevalence trends of risk factors and diseases in Northeastern Germany were evaluated. To diagnose a diabetes mellitus, in the SHIP Trend cohort the oGTT was conducted [182]. From the SHIP Trend cohort, an age-, sex-, and BMI-matched sample consisting of 850 patients was selected.

### Demographic and Phenotypic Data

Ethnical information, marriage status, psychosocial situation, age, and number of children were assessed in the bipolar group. Additionally, age of onset, polarity of first episode, number of episodes, rapid cycling, suicide attempts, and number of hospitalizations were recorded. Furthermore, current medication was assessed as well as information about alcohol and illegal drug use. Weight and height BMI and waist–hip ratio as well as blood pressure were measured. The demographic and phenotypic data are displayed in **Tables 1**–**6**. 27% (n = 23) of the bipolar patients fulfilled the NCEP ATP III criteria of a metabolic syndrome (MetS) (**Table 5**).

**Table 1:** Demographic data.

	Bipolar sample		SHIP Trend control sample	
	n	%	n	%
	85		850	
**Caucasian ethnicity**	79	93	N/A	
**Sex female:male**	37:48	44 *vs*. 66	370:480	44 vs. 66
**Age (years)**	44.72 +/−12.63 SD		46.50 ± 11.87 SD	

Patients were matched 1:10 to controls regarding age, sex, and BMI. N, number; SD, standard deviation; N/A, not available.

**Table 2:** Clinical phenotype bipolar patients.

Clinical Phenotype	Mean (SD)
**Age at onset (years)**	28.16 (±11.00)
**Duration of disease (years)**	16.60 (±10.71)
**Number of hospital stays**	4.12 (±4.48)
**Number of episodes**	14.61 (±13.48)
**Number of depressed episodes**	8.02 (±7.77)
**Number of manic episoded**	3.51 (±4.67)
**Number of hypomanic episodes**	4.06 (±5.88)
**Rapid cycling (yes)**	N23
**Suicidal attempt (yes)**	25
**Medication**	
Lithium	54
Carbamazepine	6
Oxcarbazepine	1
Lamotrigine	7
Valproate	20
Escitalopram	3
Paroxetine	1
Sertraline	2
Duloxetine	2
Venlafaxine	16
Reboxetine	1
Clomipramine	1
Doxepine	3
Trimipramine	1
Mirtazapine	2
Tranylcypromine	3
Agomelatine	3
Bupropion	2
Melperone	1
Amisulpride	1
Aripiprazole	7
Clozapine	3
Olanzapine	5
Quetiapine	30
Risperidone	3
Ziprasidone	1
Lorazepam	1
**Bipolar Subtype (I *vs*. II)**	68:17
**Comorbid disorders**	
Alcohol use disorder	5
Obsessive-compulsive disorder	2
ADHD	2
Nicotine use	28
Illegal drug use	2
Bulimia nervosa	1
Dissociative disorder	1
Dependent Personality Disorder	1
**Marital status**	
Married	51
Single	24
Divorced	10
**Education**	
9 years of schooling	2
13 years of schooling	3
Specialized job	53
College	9
University	17
**Current work status**	
Freelancer	4
Employed	30
Unemployed	9
Retired	31
Other	11

SD, standard deviation; N, number; ADHD, attention-deficit-/hyperactivity disorder.

**Table 3:** Anthropometric data.

Anthropometric data	Bipolar sample	SHIP Trend control sample
	Mean (SD)	Mean (SD)
**Weight (kg)**	85.03 (±16.78)	N/A
**Height (cm)**	170.85 (±8.67)	
**BMI, kg/m²**	29.15 (±5.60)	28.61 (±3.94)
**Waist circumference (cm)**	100.66 (±16.00)	N/A
**Hip circumference (cm)**	109.76 (±19.15)	
**WHR**	0.91 (±0.11)	
**Systolic blood pressure, mmHg**	125.22 (±14.96)	
**Diastolic Blood pressure, mmHg**	78.24 (±11.26)	

N, number; SD, standard deviation; BMI, body mass index; WHR, waist–hip-ratio; N/A, not available.

**Table 4:** T2D and pre-diabetes in bipolar patients and controls.

	Bipolar sample	SHIP Trendcontrol sample	p-value
	Total sample n = 85	Total samplen = 850	
	n (%)	n (%)	
**T2D**	6 (7%)	54 (6%)	0.8
**Prediabetes (all forms)**	28 (33%)	377 (44%)	**0.043**
*- IGT*	5 (18%)	111 (29%)	** 0.03**
*- IFG*	8 (29%)	301 (80%)	** 0.001**
*- HbA1c: 5.7–6. 4%*	18 (64%)	105 (28%)	**0.001**

N, number; T2D, diabetes mellitus type II; IGT, impaired glucose tolerance; IFT, impaired fasting glucose; differences between bipolar and control patients were analyzed by t-test. Level of significance was set at p-value p < 0.05; significant p-values are displayed in bold.

**Table 5:** Glucose and lipid metabolism data.

Blood results	Bipolar sample	SHIP Trend control sample	p-value
	Mean (SD)	Mean (SD)	
	number of patients	number of patients
**Fasting plasma glucose (mmol/l)**	4.96 (±0.80), n = 85	5.52 (±0.8), n = 850	**<0.001**
**Plasma glucose after 120 min (mmol/l)**	5.37 (±2.01), n = 83	6.39 (±2.2), n = 850	**<0.001**
**HbA1c, %**	5.42 (±0.44), n = 85	5.20 (±0.6), n = 850	**<0.001**
**Fasting Insulin (pmol/l)**	87.6 (±98.3), n = 83	N/A	
**Insulin after 120 min (pmol/l)**	290.0 (±362.0), n = 80		
**Triglycerides (mmol/l)**	1.6 (±1.0), n = 79		
**Cholesterol, mmol/l**	5.3 (±1.3), n = 79		
**HDL (mmol/l)**	1.4 (±0.4), n = 79		
**LDL (mmol/l)**	3.2 (±1.0), n = 79		

N, number; SD, standard deviation; HDL, high density lipoprotein; LDL, low density lipoprotein; differences between bipolar and control patients were analyzed by t-test; level of significance was set at p < 0.05; significant p-values are shown in bold.

**Table 6:** Comparison (pre-)diabetic and diabetic bipolar patients.

Parameter	Bipolar patients with T2D/pre-diabetes (SD), n = 34	Bipolar patients w/o diabetes/pre-diabetes (SD), n = 51	p-value
**Ages (years)**	48.82 (±11.99)	41.98 (±12.41)	**0.013**
**Sex f:m**	13:21	24:27	0.422
**BMI, kg/m²**	31.08 (±6.27)	27.87 (±4.75)	**0.014**
**Waist circumference**	104.85 (±15.33)	97.86 (±15.96)	**0.046**
**Wait–hip-ratio**	0.92 (±0.09)	0.907 (±0.10)	0.45
**Metabolic syndrome**	13 (38%)	10 (20%)	0.058
**Marital status**			
**Single**	11(32%)	13 (25%)	0.491
**Married**	21(62%)	30 (59%)	0.786
**Divorced**	2 (6%)	8 (16%)	0.169
**Current work status**			
**Employed/freelancer**	11 (32%)	24 (47%)	0.14
**Pensioned**	17 (50%)	14 (27%)	
**Unemployed**	3 (9%)	6 (12%)	
**Other**	3 (9%)	7 (14%)	
**Age of onset**	28.91 (±10.49)	27.67 (±11.40)	0.61
**Disease duration**	20.0 (±11.14)	14.33 (±9.89)	**0.019**
**Number of depressive episodes**	10.91 (±9.10)	6.10 (±6.11)	**0.01**
**Number of manic episodes**	3.18 (±4.32)	3.31 (±4.90)	0.83
**Number of hypomanic episodes**	3.18 (±4.95)	2.22 (±5.20)	0.40
**Number of mixed episodes**	0.65 (±0.88)	0.67(±1.66)	0.95
**Total number of episodes**	17.91 (±14.33)	12.24 (±12.26)	0.054
**Episodes per disorder year**	0.9	0.85	0.485
**Rapid Cycling**	12 (35%)	11 (22%)	0.163
**Sucidal attempts**	9 (26%)	16 (31%)	0.627
**Number of hospitalizations**	4.03 (±3.49)	4.18 (±5.06)	0.883
**FINDRISK-Score**	10.91 (±4.98)	7.69 (±4.02)	**0.003**
**WHO-5 Score**	13.53 (±5.97)	14.65 (±5.00)	0.353
**SF-12 Score**	30.09 (±3.97)	31.33 (±2.21)	0.067

N, number; SD, standard deviation; differences between diabetic and nondiabetic bipolar patients were calculated by t-test or χ^2^ test, respectively. Level of significance was set at p < 0.05. Significant p-values are marked in bold.

### Oral Glucose Tolerance Test

The oral glucose tolerance test (oGTT) is a standardized test and validated diagnostic instrument in the clinical routine to verify the diagnosis of a diabetes mellitus and an impaired glucose tolerance (IGT) (38). The test was conducted following the WHO guidelines (38). Three days before the test, the patients refrained from their usual diet. 10 h before the test patients fasted (including food, alcohol, coffee, and increased activity). The oGTT was conducted between 8 and 11 am, and the patients did not take their medication directly before the test. The oGTT was not conducted three days before, during or 3 days after the menstrual bleeding. Venous blood was taken, and plasma glucose, insulin, and lipid levels were measured at fasting baseline. After that the patients ingested 75 g glucose dissolved in 300 ml water (*Roche Dextro OGT, Basel, Switzerland*). They were instructed to drink it in 5 min. After 120 min venous blood was drawn for the second time. Glucose was measured from the venous plasma collected in a fluoride tube which inhibits glycolysis. HBA1c was measured from blood collected in an EDTA tube, and lipids were measured from blood in serum tubes (total cholesterol, high-density cholesterol, low-density cholesterol, triglycerides). The analyses were conducted in the central clinical routine laboratories of the University Hospitals of Dresden and Würzburg.

### Questionnaires

#### SF-12

The patients were evaluated regarding their health-related quality of life by using the SF-12 questionnaire. This is a short form of the SF-36-health questionnaire and includes eight dimensions (body functioning, bodily role function, pain, general assessment of health, vitality, social functioning, emotional role function, mental well-being) to measure the cross-disorder health-related quality of life during the past 4 weeks (41).

#### WHO-5

WHO-5 is a questionnaire for evaluating well-being. There are five questions that cover the dimensions mood, vitality and general interest during the past 2 weeks. A Likert scale is used from 0 (= never) to 5 (= always) and a sum score can be calculated with values between 0 and 25 (42). The WHO-5-questionnaire is recommended as a screening instrument for depression for example in patients with T1D and T2D (43).

#### Finnish Diabetes Risk Score Questionnaire

The *Finnish Diabetes Risk Score Questionnaire* (FINDRISC) was developed as a risk assessment to effectively prevent T2D in Finland (44). To use this instrument in Germany, a modified version (due to different life styles and eating habits) was developed (FINDRISK). Age, family history of diabetes mellitus, waist circumference, activity level, eating habits, arterial hypertension, increased blood glucose levels in the past, and body mass index are assessed. The sum score is 0 to 26 (45).

#### Montgomery–Åsberg Depression Rating Scale (MADRS)

The MADRS is a structured interview for the quantitative assessment of depressive symptoms severity. The maximum sum score is 60. It can be conducted evaluating the last 24 h or the last week (46).

#### Young Mania Rating Scale

The YMRS is a structured interview for the quantitative assessment of manic symptom severity during the last 48 h (47).

#### Clinical Global Impressions Scale for Bipolar Disorder

The CGI-BP is a scale to assess the clinical severity in bipolar affective disorder. The scale combines separate items for mania, depression and global impression of the bipolar patient. The severity of the disorder can be scored from 1 (not ill) to 7 (severely ill) (48).

#### Statistical Analysis

Prevalence of T2D and prediabetes in the SHIP-Trend- and BiDi-samples as well as the results of the questionnaires and anthropometric measurements were assessed by descriptive statistics using SPSS (IBM® SPSS® Statistics 20). Data were tested for normal distribution and differences between groups were tested by χ²-test and t-test. Furthermore, we investigated the correlation of metabolic parameters with the number of disease episode in the bipolar patients by Pearson’s correlation test. Additionally, as a secondary analysis, a multivariate analysis was calculated to investigate differences in the multiple variates between the nondiabetic and (pre-)diabetic bipolar groups (MANOVA). The level of significance was set at p =< 0.05.

## Results

### Prevalence of Diabetes Mellitus Type II and Prediabetes in Patients Suffering From BD *vs*. General Population

**Figure 1:**
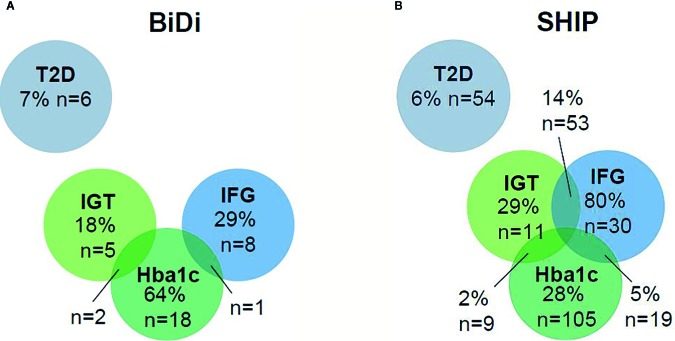
**(A, B)** T2D and prediabetes rates in bipolar patients and controls.

We diagnosed T2D and prediabetes by using the oGTT results and the HbA1c-value (according to ADA-criteria) (38). In the patient sample, 7% of the patients fulfilled the diagnostic criteria of T2D. In two of those cases, T2D had already been diagnosed before. In the SHIP Trend control cohort, 6% of the control participants fulfilled the diagnostic criteria of T2D. The difference in T2D prevalence between BiDi and SHIP-Trend control group was not statistically significant (χ^2^ = 0.064, p = 0.8) (**Table 4**, **Figures 1A, B**).

Prediabetes could be diagnosed in 33% of the bipolar patients *vs.* 44% of the controls. In 18% of the prediabetic bipolar patients, impaired glucose tolerance (IGT) could be determined *vs*. 29% of the controls. 29% of the bipolar prediabetic patients had an impaired fasting glucose (IFG) *vs*. 80% of the prediabetic controls. 64% of the prediabetic bipolar patients showed an HbA1c value in the prediabetic range *vs*. 28% of the prediabetic controls. In 14% of the control individuals, IFG and IGT occurred simultaneously. 10% of the control participants had an increased HbA1c and IFG, whereas 2% had an increased HbA1c value and IGT. In 5% of the control population, all parameters were in the prediabetic range. Furthermore, IGT and an increased HbA1c value were found in7% of the control participants. 4% had IFG and an increased HbA1c value (**Table 4**, **Figures 1A, B**). None of the participants showed both IGT and IFG. Prediabetes was significantly more common in the control group in comparison to the bipolar sample (χ^2^ = 4.106, p = 0.043).

Comparison of the fasting glucose levels of both groups showed that BD patients had significantly lower glucose levels than control participants [t(933) = 6.395, p = <0.001, see **Table 5**]. Additionally, blood glucose levels after 120 min in the oGTT were significantly lower in BD patients than in control participants [t(931) = 4.056, p = <0.001, see **Table 5**]. However, HbA1c values of the bipolar sample were significantly higher in comparison to the SHIP Trend control population [t(933) = −3.234, p = <0.001).

Here, the rates of diabetes mellitus type II (T2D) and prediabetic conditions (IGT, impaired glucose tolerance; IFG, impaired fasting glucose) as well as HbA1c values are displayed in a Venn diagram for the bipolar sample (BiDi) and the age-, sex- and BMI-matched sample from the general population (SHIP).

### Comparison of the Prediabetic/Diabetic BD Patients *vs*. Nondiabetic Bipolar Patients

To investigate the risk factors for diabetes in the sample of bipolar patients, we compared the prediabetic and diabetic bipolar patients with the nondiabetic bipolar patients. The prediabetic/diabetic bipolar patients were significantly older and had a significantly longer disease duration, had a significantly higher BMI and waist circumference in comparison to the nondiabetic bipolar patients (**Table 5**). Furthermore, they have had suffered from a higher number of depressive episodes and had significantly higher scores in the FINDRISK. The other variables were not significantly different between the groups (**Table 6**). Additionally, there was no significant difference in the medication between the diabetic and nondiabetic bipolar groups (lithium carbonate: χ² = 1,430, p = 0.232; valproate: χ² = 2.452, p = 0.117; quetiapine: χ² = 0.859, p = 0.354). There was also no difference in the distribution between patients taking olanzapine and clozapine as drugs with a potential high metabolic risk and patients taking aripiprazole as a drug with a potential protective effect against diabetes between the nondiabetic and the (pre-)diabetic group (χ² = 1,122, p = 0.571). Furthermore, there was a significant positive correlation between the blood glucose levels, HbA1c and BMI, and the number of depressive episodes, but not manic or mixed episodes (see **Table 7**). We additionally conducted a multivariate analysis (MANOVA) to investigate group differences between the nondiabetic and the (pre-)diabetic group. We were taking age, BMI, disease duration, and number of depressive episodes into account as covariates. All those variates (age, BMI, number of episodes, disease duration) remained significantly different between the bipolar groups in the multivariate analysis (p = 0.006 and between subject effects were for age p = 0.013, for BMI p = 0.009, for depressive episodes p = 0.004 and for disease duration p = 0.016, respectively).

**Table 7:** Correlation of metabolic parameters with disorder severity.

		Fasting plasma glucose (mmol/l)	Plasma glucose after 120 min (mmol/l)	HbA1c, %	BMI, kg/m^2^
**Number of depressed episodes**	Pearson Correlation	,245^*^	0.212	,428^**^	,306^**^
	Sig. (2-tailed)	**0.024**	**0.051**	**0.0001**	**0.004**
**Number of manic episodes**	Pearson Correlation	0.052	0.078	0.147	0.163
	Sig. (2-tailed)	0.634	0.476	0.180	0.137
**Number of hypomanic episodes**	Pearson Correlation	0.059	0.014	0.177	0.059
	Sig. (2-tailed)	0.593	0.896	0.107	0.595
**Number of mixed episodes**	Pearson Correlation	0.022	0.095	−0.015	,240^*^
	Sig. (2-tailed)	0.842	0.387	0.894	**0.027**
**Number of all episodes**	Pearson Correlation	0.185	0.167	,368^**^	,284^**^
	Sig. (2-tailed)	0.090	0.128	**0.001**	**0.008**
**Disease duration**	Pearson Correlation	,323^**^	0.170	,433^**^	,243^*^
	Sig. (2-tailed)	**0.003**	0.119	**0.0001**	**0.025**

Pearson’s correlation test was conducted. **. Correlation is significant at the 0.01 level (2-tailed). *. Correlation is significant at the 0.05 level (2-tailed). Level of significance was set at p = 0.05, significant p-values are shown in bold. Number of bipolar patients included in the analysis was 85.

## Discussion

In our study, we could not find an increased rate of T2D in bipolar patients in comparison to age-, sex- and BMI-matched controls. These findings are in contrast to previous studies reporting increased prevalence of T2D in bipolar patients (9–12). When we restricted the analysis of our data to nonmatched controls and used the older (2006) instead of the newer ADA-criteria (49), our previously published report also suggested an increased rate of diabetes and prediabetes in BD in line with previous studies (36). However, when comparing the parameters directly to an age-, sex- and BMI-matched matched control population, we could no longer find any difference in T2D rates and even lower rates of prediabetes and lower levels of fasting glucose as well as oGTT values in BD. Only the HbA1c values were significantly higher in our bipolar sample compared to the SHIP-Trend general population sample although the effect size was rather small (Cohen’s d = 0.037).

Our main finding, *i.e.* that the rate of T2D in BD is not increased, in comparison to a general population sample might well be due to the fact that we used age-, sex- and BMI-matched controls. Obesity is a major risk factor for T2D (50) and is also positively associated with BD (OR = 1.77, 95% CI: 1.40–2.23; Q = 44.62, P < 0.001) (51, 52). Therefore, we speculate that the increased risk for T2D in other studies might be a consequence of significantly increased rates of obesity in the BD compared to the general population. In comparison with another German general population study [*Studie zur Gesundheit Erwachsener in Deutschland* (DEGS1, 2008–2011)] our BiDi group with a mean BMI of 29.15 had a significantly higher BMI as the age-stratified controls (53). In the DEGS1 cohort, 67% of the men and 53% of the females in the same age range as our BiDi sample had a BMI >= 25. In our BiDi sample, 78% of the participants had a BMI > 25. Taken together, we propose that overweight and obesity might be the mediating factors between BD and T2D, and that the risk for T2D in BD in comparison to the general population may not be increased in BD as such but rather the risk towards obesity. A previous Italian study and a follow-up study could also show that abdominal obesity as a major factor of the metabolic syndrome was associated with a higher rate of T2D in bipolar patients (54, 55). The higher rate of obesity can be due to either lifestyle factors (food pattern, sedentary lifestyle), medication influence (especially second-generation antipsychotics such as olanzapine and quetiapine) (56) as well as shared risk genes for BD and BMI (57). However, as a limiting factor in our study, we did not include other factors that, especially in men, have shown to increase the risk of T2D like smoking and arterial hypertension (58, 59). In our sample, there was however no significant difference between the types of mood stabilizing medication in the diabetic *vs*. the nondiabetic group. But then, also valproate and lithium can lead to weight gain and not only atypical antipsychotics (19, 60). Also, our sample size in the medication subgroups was too small to make definite conclusions hereon. Interestingly, in a multivariate analysis comparing the nondiabetic and (pre-)diabetic bipolar groups, disease duration and number of depressive episodes, as well as BMI and age, remained statistically significant between the groups. Furthermore, metabolic parameters were significantly correlated with the number of depressed episodes. There are previous studies that suggest that comorbid insulin resistance, diabetes mellitus type **II,** and an increased BMI might lead to a more severe course in bipolar patients (61–63). From our data, we might conclude that increased BMI is the major contributor to an increased risk for T2D in BD in comparison to the general population; however, disease duration and depressive polarity might add to the risk of developing T2D in BD patients. However, as our study was a cross-sectional and not a longitudinal study, we cannot confirm the direction of the association of impaired metabolic parameters and a more severe course. Diagnosing and monitoring of overweight and prediabetes and T2DM in BD are furthermore of importance as there is growing evidence that impaired glucose metabolism and T2D might lead to worse response to treatment with mood stabilizers (64).

As being overweight is a modifiable risk state, special emphasis should be paid to lifestyle modification in BD patients to avoid detrimental general health outcomes including T2D. Unhealthy lifestyle that increases the risk of obesity seems more to be an issue of depressive episodes than manic episodes supposedly due to lack of activity and unhealthy eating patterns. However, as we did not assess information about activity and diet in association with mood episodes, we only can speculate about this. In a pilot study investigating the effectiveness of lifestyle interventions to reduce glucometabolic risks in BD, there were positive preliminary results (65). Several associations and societies recommend metabolic monitoring in patients taking second-generation antipsychotic drugs; however this is not yet implemented fully in clinical routine (38, 66). We here strongly recommend routing monitoring of at least noninvasive anthropometric measures such as BMI and WHR to detect weight increase early on and to take appropriate measures.

Several previous studies have pointed towards an increased prevalence of T2D in BD. For example, Cassidy and colleagues reported a prevalence of T2D in bipolar patients of 9.9% which was significantly higher than the 3.4% diabetes mellitus rate in the control population in their study (12). Lilliker et al. described that 10% of BD patients suffer from diabetes as compared to 2% in their non-bipolar sample (9). Regenold et al. found the T2D prevalence as high as 26% in bipolar-I patients, compared to 13% in the control population (11). In a Belgium sample, diabetes was prevalent in 6.7% of the bipolar group which was twice as often as in the age-matched control group (10). The main reason for the wide range of prevalence rates in bipolar sample between 6.7%, which is similar to our sample, and 26% most likely lies in the different mean age of the various samples. Age is a validated risk factor of T2D, the higher the age, the higher the prevalence of T2D, especially from the age of 50 years on (67). The lowest prevalence rates were accordingly found in the samples with lower mean age, as it was the case in our sample with 7% T2D prevalence with a mean age of 44.7 years. The bipolar sample from Belgium with a 6.7% T2D included bipolar patients with a mean age of 42.1 years, and the sample of Cassidy et al., with a prevalence of 9.9%, had a mean age of 45.3 years (10, 12). In line with this, patients suffering from both BD and T2D in our sample also were significantly older than the nondiabetic bipolar patients. Another reason for differing T2D prevalence might be due to diagnostic and assessment procedures. The majority of studies used information based on the hospital medical records (9, 11, 12), only van Winkel and colleagues validated the diagnosis by using the oGTT, as done in our study (10). Due to the large number of undiagnosed T2D cases in the general population, prevalence rates that only rely on self-report or medical records might be too low. The *Kora F4 Survey* showed a prevalence of previously undiagnosed T2D of 2.0% in addition to the already known T2D of 2.2% in the general population aged between 35 and 59 years using the oGTT (68). Also other studies estimate about 50% existing but undiagnosed T2D cases in the general population worldwide (69).

Another notable difference between our and the other studies is that we enrolled only euthymic patients. In contrast to Regenold et al., van Winkel et al., and Lilliker et al., we only included patients who were euthymic and on stable medication for at least 2 months, as the metabolic status might be influenced in acute episodes.

A systematic review and meta-analysis investigating the prevalence of diabetes mellitus in BD, schizophrenia, and major depression reported a mean T2D prevalence of 9.4% in BD (70). In comparison to age- and sex-adjusted control population, this was a relative risk of 1.89 in patients with BD (n = 54,688; 95% confidence interval: 1.29–2.77, p < 0.001). The authors of this review came to the conclusion that there were also quite large geographical differences in T2D prevalence in the general population which need to be taken into account when comparing bipolar patients and the general population (70). In our sample, there were controls mainly from the northeastern part of Germany compared to a mainly southern sample of bipolar patients. However, the diabetes mellitus prevalence between north and south of Germany has shown to be very similar in the recent years (71).

In conclusion, we could not find an increased prevalence of prediabetes and T2D in BD when an age-, sex- and BMI-matched control sample is used in comparison. Comparing diabetic and nondiabetic bipolar patients, we could identify disease duration and more depressive episodes as potential risk factors for developing T2D or potentially strengthen previous findings of comorbid (pre-)diabetes mellitus as risk factor for a more severe course of the disorder. The direction of interaction we could not determine due to the cross-sectional character of our study. The FINDRISK score was significantly higher in the prediabetic and T2D bipolar patients which strengthens the validity for a screening tool also in patient populations. We hypothesize that obesity might be mediating the previously reported increased T2DM prevalence in BD in comparison to the general population, and medication side effects might contribute to this as well as a longer duration of the disorder and depressive polarity. However, due to the small number of patients in the subgroups, we could not determine differential effects of the different mood stabilizing drugs. Adequate weight and metabolic monitoring and intervention may lead to improved outcomes in this patient group, which is especially important given the reduced life expectancy of patients with BD due to somatic disorders (7).

## Data Availability Statement

The datasets generated for this study are available on request to the corresponding author.

## Ethics Statement

The studies involving human participants were reviewed and approved by the Ethics Committees of the Universities of Würzburg, Dresden and Greifswald. The patients/participants provided their written informed consent to participate in this study.

## Author Contributions

SK-S, AR, and DB recruited the patients and collected the sample. DB collected the phenotypic data, drew the blood, and analyzed the data. SK-S wrote the paper draft. AR took part in writing and revising the final manuscript. SH and AP designed the study. SH and KL recruited the patients and collected the samples in the study center Dresden. SP built and managed the data base. SP and CS checked the data quality in the study center Dresden. AP and MB supervised the study management in the study center Dresden and critically reviewed the manuscript. H-JG and HV provided the data of the SHIP cohorts.

## Funding

This study has been supported by a grant of the medical faculty of the Technische Universität Dresden to SH and AP. This publication was funded by the University of Frankfurt. SHIP is part of the Community Medicine Research net of the University of Greifswald, Germany, which is funded by the Federal Ministry of Education and Research (grants no. 01ZZ9603, 01ZZ0103, and 01ZZ0403), the Ministry of Cultural Affairs and the Social Ministry of the Federal State of Mecklenburg-West Pomerania.

## Conflict of Interest

SK-S has received speaker’s and author’s honoraria from Medice and Takeda. AR has received speaker fees and honoraria (publications, advisory boards) from Medice, Shire/Takeda, Servier, neuraxpharm, Janssen, and SAGE. KL has been an advisor to and received speaker’s honoraria and travel support from Janssen, Lundbeck, Otsuka and Recordati. She received grant support from Janssen and Otsuka. AP has received speaker’s honoraria and travel support from Janssen and Lundbeck. MB has been an advisor to Janssen-Cilag, neuraxpharm und Sunovion, and received speaker’s honoraria from Hexal AG, Janssen-Cilag und Sunovion. H-JG has received travel grants and speaker’s honoraria from Fresenius Medical Care, Neuraxpharm, and Janssen Cilag as well as research funding from Fresenius Medical Care.

The remaining authors declare that the research was conducted in the absence of any commercial or financial relationships that could be construed as a potential conflict of interest.

## References

[1] Newcomer JW Medical risk in patients with bipolar disorder and schizophrenia. J Clin Psychiatry (2006) 67(11):e16. 10.4088/JCP.1106e16 17201046

[2] Carney CP Jones LE Medical comorbidity in women and men with bipolar disorders: a population-based controlled study. Psychosom Med (2006) 68(5):684–91. 10.1097/01.psy.0000237316.09601.88 17012521

[3] Walker ER McGee RE Druss BG Mortality in mental disorders and global disease burden implications: a systematic review and meta-analysis. JAMA Psychiatry (2015) 72(4):334–41. 10.1001/jamapsychiatry.2014.2502 PMC4461039 25671328

[4] Khan A Faucett J Morrison S Brown WA Comparative mortality risk in adult patients with schizophrenia, depression, bipolar disorder, anxiety disorders, and attention-deficit/hyperactivity disorder participating in psychopharmacology clinical trials. JAMA Psychiatry (2013) 70(10):1091–9. 10.1001/jamapsychiatry.2013.149 23986353

[5] Hayes JF Marston L Walters K King MB Osborn DPJ Mortality gap for people with bipolar disorder and schizophrenia: UK-based cohort study 2000-2014. Br J Psychiatry (2017) 211(3):175–81. 10.1192/bjp.bp.117.202606 PMC5579328 28684403

[6] Correll CU Solmi M Veronese N Bortolato B Rosson S Santonastaso P Prevalence, incidence and mortality from cardiovascular disease in patients with pooled and specific severe mental illness: a large-scale meta-analysis of 3,211,768 patients and 113,383,368 controls. World Psychiatry (2017) 16(2):163–80. 10.1002/wps.20420 PMC5428179 28498599

[7] Schneider F Erhart M Hewer W Loeffler LA Jacobi F Mortality and Medical Comorbidity in the Severely Mentally Ill. Dtsch Arztebl Int (2019) 116(23-24):405–11. 10.3238/arztebl.2019.0405 PMC6683445 31366432

[8] Charles EF Lambert CG Kerner B Bipolar disorder and diabetes mellitus: evidence for disease-modifying effects and treatment implications. Int J Bipolar Disord (2016) 4(1):13. 10.1186/s40345-016-0054-4 27389787 PMC4936996

[9] Lilliker SL Prevalence of diabetes in a manic-depressive population. Compr Psychiatry (1980) 21(4):270–5. 10.1016/0010-440X(80)90030-9 7398250

[10] van Winkel R De Hert M Van Eyck D Hanssens L Wampers M Scheen A Prevalence of diabetes and the metabolic syndrome in a sample of patients with bipolar disorder. Bipolar Disord (2008) 10(2):342–8. 10.1111/j.1399-5618.2007.00520.x 18271914

[11] Regenold WT Thapar RK Marano C Gavirneni S Kondapavuluru PV Increased prevalence of type 2 diabetes mellitus among psychiatric inpatients with bipolar I affective and schizoaffective disorders independent of psychotropic drug use. J Affect Disord (2002) 70(1):19–26. 10.1016/S0165-0327(01)00456-6 12113916

[12] Cassidy F Ahearn E Carroll BJ Elevated frequency of diabetes mellitus in hospitalized manic-depressive patients. Am J Psychiatry (1999) 156(9):1417–20. 10.1176/ajp.156.9.1417 10484954

[13] McIntyre RS Konarski JZ Misener VL Kennedy SH Bipolar disorder and diabetes mellitus: epidemiology, etiology, and treatment implications. Ann Clin Psychiatry (2005) 17(2):83–93. 10.1080/10401230590932380 16075661

[14] Lustman PJ Griffith LS Clouse RE Cryer PE Psychiatric illness in diabetes mellitus. Relationship to symptoms and glucose control. J Nerv Ment Dis (1986) 174(12):736–42. 10.1097/00005053-198612000-00005 3783141

[15] Guo JJ Keck PE Jr. Corey-Lisle PK Li H Jiang D Jang R Risk of diabetes mellitus associated with atypical antipsychotic use among patients with bipolar disorder: A retrospective, population-based, case-control study. J Clin Psychiatry (2006) 67(7):1055–61. 10.4088/JCP.v67n0707 16889448

[16] Haupt DW Newcomer JW Abnormalities in glucose regulation associated with mental illness and treatment. J Psychosom Res (2002) 53(4):925–33. 10.1016/S0022-3999(02)00471-3 12377305

[17] Yood MU DeLorenze G Quesenberry CP Jr. Oliveria SA Tsai AL Willey VJ The incidence of diabetes in atypical antipsychotic users differs according to agent–results from a multisite epidemiologic study. Pharmacoepidemiol Drug Saf (2009) 18(9):791–9. 10.1002/pds.1781 19526626

[18] Keck PE McElroy SL Bipolar disorder, obesity, and pharmacotherapy-associated weight gain. J Clin Psychiatry (2003) 64(12):1426–35. 10.4088/JCP.v64n1205 14728103

[19] Torrent C Amann B Sanchez-Moreno J Colom F Reinares M Comes M Weight gain in bipolar disorder: pharmacological treatment as a contributing factor. Acta Psychiatr Scand (2008) 118(1):4–18. 10.1111/j.1600-0447.2008.01204.x 18498432

[20] Taylor V MacQueen G Associations between bipolar disorder and metabolic syndrome: A review. J Clin Psychiatry (2006) 67(7):1034–41. 10.4088/JCP.v67n0704 16889445

[21] Kajio Y Kondo K Saito T Iwayama Y Aleksic B Yamada K Genetic association study between the detected risk variants based upon type II diabetes GWAS and psychotic disorders in the Japanese population. J Hum Genet (2014) 59(1):54–6. 10.1038/jhg.2013.116 24196380

[22] Torkamani A Topol EJ Schork NJ Pathway analysis of seven common diseases assessed by genome-wide association. Genomics. (2008) 92(5):265–72. 10.1016/j.ygeno.2008.07.011 PMC2602835 18722519

[23] Stahl EA Breen G Forstner AJ McQuillin A Ripke S Trubetskoy V Genome-wide association study identifies 30 loci associated with bipolar disorder. Nat Genet (2019) 51(5):793–803. 10.1038/s41588-019-0397-8 31043756 PMC6956732

[24] Bent-Ennakhil N Cecile Perier M Sobocki P Gothefors D Johansson G Milea D Incidence of cardiovascular diseases and type-2-diabetes mellitus in patients with psychiatric disorders. Nord J Psychiatry (2018) 72(7):455–61. 10.1080/08039488.2018.1463392 30513230

[25] Kember RL Hou L Ji X Andersen LH Ghorai A Estrella LN Genetic pleiotropy between mood disorders, metabolic, and endocrine traits in a multigenerational pedigree. Transl Psychiatry (2018) 8(1):218. 10.1038/s41398-018-0226-3 30315151 PMC6185949

[26] Elmslie JL Mann JI Silverstone JT Williams SM Romans SE Determinants of overweight and obesity in patients with bipolar disorder. J Clin Psychiatry (2001) 62(6):486–91; quiz 92-3. 10.4088/JCP.v62n0614 11465534

[27] Kilbourne AM Rofey DL McCarthy JF Post EP Welsh D Blow FC Nutrition and exercise behavior among patients with bipolar disorder. Bipolar Disord (2007) 9(5):443–52. 10.1111/j.1399-5618.2007.00386.x 17680914

[28] Lasser K Boyd JW Woolhandler S Himmelstein DU McCormick D Bor DH Smoking and mental illness: A population-based prevalence study. JAMA. (2000) 284(20):2606–10. 10.1001/jama.284.20.2606 11086367

[29] Garcia-Portilla MP Saiz PA Benabarre A Florez G Bascaran MT Diaz EM Impact of substance use on the physical health of patients with bipolar disorder. Acta Psychiatr Scand (2010) 121(6):437–45. 10.1111/j.1600-0447.2009.01498.x 19895620

[30] Chengappa KN Levine J Gershon S Kupfer DJ Lifetime prevalence of substance or alcohol abuse and dependence among subjects with bipolar I and II disorders in a voluntary registry. Bipolar Disord (2000) 2(3 Pt 1):191–5. 10.1034/j.1399-5618.2000.020306.x 11256686

[31] Waxmonsky JA Thomas MR Miklowitz DJ Allen MH Wisniewski SR Zhang H Prevalence and correlates of tobacco use in bipolar disorder: data from the first 2000 participants in the Systematic Treatment Enhancement Program. Gen Hosp Psychiatry (2005) 27(5):321–8. 10.1016/j.genhosppsych.2005.05.003 16168792

[32] Aas M Bellivier F Bettella F Henry C Gard S Kahn JP Childhood maltreatment and polygenic risk in bipolar disorders. Bipolar Disord (2019). 22(2):174–81. 10.1111/bdi.12851 31628696

[33] Huffhines L Noser A Patton SR The Link Between Adverse Childhood Experiences and Diabetes. Curr Diabetes Rep (2016) 16(6):54. 10.1007/s11892-016-0740-8 PMC5292871 27112958

[34] Soczynska JK Kennedy SH Woldeyohannes HO Liauw SS Alsuwaidan M Yim CY Mood disorders and obesity: understanding inflammation as a pathophysiological nexus. Neuromol Med (2011) 13(2):93–116. 10.1007/s12017-010-8140-8 21165712

[35] McIntyre RS Danilewitz M Liauw SS Kemp DE Nguyen HT Kahn LS Bipolar disorder and metabolic syndrome: an international perspective. J Affect Disord (2010) 126(3):366–87. 10.1016/j.jad.2010.04.012 20541810

[36] Leopold K Reif A Haack S Bauer M Bury D Loffler A Type 2 diabetes and pre-diabetic abnormalities in patients with bipolar disorders. J Affect Disord (2016) 189:240–5. 10.1016/j.jad.2015.09.041 26451510

[37] Sievers C Auer MK Klotsche J Athanasoulia AP Schneider HJ Nauck M IGF-I levels and depressive disorders: results from the Study of Health in Pomerania (SHIP). Eur Neuropsychopharmacol (2014) 24(6):890–6. 10.1016/j.euroneuro.2014.01.008 24507017

[38] American Diabetes A Diagnosis and classification of diabetes mellitus. Diabetes Care (2010) 33 Suppl 1:S62–9. 10.2337/dc10-S062 PMC2797383 20042775

[39] Block A Schipf S Van der Auwera S Hannemann A Nauck M John U Sex- and age-specific associations between major depressive disorder and metabolic syndrome in two general population samples in Germany. Nord J Psychiatry (2016) 70(8):611–20. 10.1080/08039488.2016.1191535 27299922

[40] Volzke H Alte D Schmidt CO Radke D Lorbeer R Friedrich N Cohort profile: the study of health in Pomerania. Int J Epidemiol (2011) 40(2):294–307. 10.1093/ije/dyp394 20167617

[41] Ware J Jr. Kosinski M Keller SD A 12-Item Short-Form Health Survey: construction of scales and preliminary tests of reliability and validity. Med Care (1996) 34(3):220–33. 10.1097/00005650-199603000-00003 8628042

[42] Topp CW Ostergaard SD Sondergaard S Bech P The WHO-5 Well-Being Index: a systematic review of the literature. Psychother Psychosom (2015) 84(3):167–76. 10.1159/000376585 25831962

[43] Hajos TR Pouwer F Skovlund SE Den Oudsten BL Geelhoed-Duijvestijn PH Tack CJ Psychometric and screening properties of the WHO-5 well-being index in adult outpatients with Type 1 or Type 2 diabetes mellitus. Diabetes Med (2013) 30(2):e63–9. 10.1111/dme.12040 23072401

[44] Salinero-Fort MA Carrillo-de Santa Pau E Abanades-Herranz JC Dujovne-Kohan I Cardenas-Valladolid J en nombre del Grupo M [Baseline risk of Diabetes Mellitus in Primary Health Care Services by FINDRISC test, associated factors and clinical outcome after 18 months of follow-up]. Rev Clin Esp (2010) 210(9):448–53. 10.1016/j.rce.2010.03.008 20667531

[45] Schwarz PE Schuppenies A Gruhl U Hoffmann R Bornstein SR Schulze J [Prevention of type 2 diabetes in Germany. Ideas, evidence, implementation]. Med Klin (Munich) (2006) 101(9):730–6. 10.1007/s00063-006-1100-2 16977398

[46] Schmidtke A Fleckenstein P Moises W Beckmann H [Studies of the reliability and validity of the German version of the Montgomery-Asberg Depression Rating Scale (MADRS)]. Schweiz Arch Neurol Psychiatr (1988) 139(2):51–65. 2455937

[47] Muhlbacher M Egger C Kaplan P Simhandl C Grunze H Geretsegger C [Reliability and concordance validity of a German version of the Young Mania Rating Scale (YMRS-D)]. Neuropsychiatr (2011) 25(1):16–25. 21486540

[48] Spearing MK Post RM Leverich GS Brandt D Nolen W Modification of the Clinical Global Impressions (CGI) Scale for use in bipolar illness (BP): the CGI-BP. Psychiatry Res (1997) 73(3):159–71. 10.1016/S0165-1781(97)00123-6 9481807

[49] American Diabetes A Standards of medical care in diabetes–2013. Diabetes Care (2013) 36 Suppl 1:S11–66. 10.2337/dc13-S011 PMC3537269 23264422

[50] Kahn SE Hull RL Utzschneider KM Mechanisms linking obesity to insulin resistance and type 2 diabetes. Nature. (2006) 444(7121):840–6. 10.1038/nature05482 17167471

[51] Zhao Z Okusaga OO Quevedo J Soares JC Teixeira AL The potential association between obesity and bipolar disorder: A meta-analysis. J Affect Disord (2016) 202:120–3. 10.1016/j.jad.2016.05.059 27262632

[52] Barton BB Zagler A Engl K Rihs L Musil R Prevalence of obesity, metabolic syndrome, diabetes and risk of cardiovascular disease in a psychiatric inpatient sample: results of the Metabolism in Psychiatry (MiP) Study. Eur Arch Psychiatry Clin Neurosci (2019). 10.1007/s00406-019-01043-8 31302731

[53] Mensink GB Schienkiewitz A Haftenberger M Lampert T Ziese T Scheidt-Nave C [Overweight and obesity in Germany: results of the German Health Interview and Examination Survey for Adults (DEGS1)]. Bundesgesundheitsblatt Gesundheitsforschung Gesundheitsschutz. (2013), 56(5–6)786–94. 10.1007/s00103-012-1656-3 23703499

[54] Salvi V D’Ambrosio V Rosso G Bogetto F Maina G Age-specific prevalence of metabolic syndrome in Italian patients with bipolar disorder. Psychiatry Clin Neurosci (2011) 65(1):47–54. 10.1111/j.1440-1819.2010.02160.x 21265935

[55] Salvi V D’Ambrosio V Bogetto F Maina G Metabolic syndrome in Italian patients with bipolar disorder: a 2-year follow-up study. J Affect Disord (2012) 136(3):599–603. 10.1016/j.jad.2011.10.025 22119084

[56] Schneider-Thoma J Efthimiou O Bighelli I Dorries C Huhn M Krause M Second-generation antipsychotic drugs and short-term somatic serious adverse events: a systematic review and meta-analysis. Lancet Psychiatry (2019) 6(9):753–65. 10.1016/S2215-0366(19)30223-8 31320283

[57] Bahrami S Steen NE Shadrin A O’Connell K Frei O Bettella F Shared Genetic Loci Between Body Mass Index and Major Psychiatric Disorders: A Genome-wide Association Study. JAMA Psychiatry (2020). 10.1001/jamapsychiatry.2019.4188 PMC6990967 31913414

[58] Yuan S Larsson SC A causal relationship between cigarette smoking and type 2 diabetes mellitus: A Mendelian randomization study. Sci Rep (2019) 9(1):19342. 10.1038/s41598-019-56014-9 31852999 PMC6920406

[59] Yang F Ma Q Liu J Ma B Guo M Liu F Prevalence and major risk factors of type 2 diabetes mellitus among adult psychiatric inpatients from 2005 to 2018 in Beijing, China: a longitudinal observational study. BMJ Open Diabetes Res Care (2020) 8(1):e000996. 10.1136/bmjdrc-2019-000996 PMC7059541 32139600

[60] Bowden CL Valproate. Bipolar Disord (2003) 5(3):189–202. 10.1034/j.1399-5618.2003.00031.x 12780873

[61] Cairns K McCarvill T Ruzickova M Calkin CV Course of bipolar illness worsens after onset of insulin resistance. J Psychiatr Res (2018) 102:34–7. 10.1016/j.jpsychires.2018.03.006 29579625

[62] Calkin CV Ruzickova M Uher R Hajek T Slaney CM Garnham JS Insulin resistance and outcome in bipolar disorder. Br J Psychiatry (2015) 206(1):52–7. 10.1192/bjp.bp.114.152850 25323142

[63] Calkin C van de Velde C Ruzickova M Slaney C Garnham J Hajek T Can body mass index help predict outcome in patients with bipolar disorder? Bipolar Disord (2009) 11(6):650–6. 10.1111/j.1399-5618.2009.00730.x PMC3544930 19689507

[64] Steardo L Jr. Fabrazzo M Sampogna G Monteleone AM D’Agostino G Monteleone P Impaired glucose metabolism in bipolar patients and response to mood stabilizer treatments. J Affect Disord (2019) 245:174–9. 10.1016/j.jad.2018.10.360 30391773

[65] Sylvia LG Salcedo S Bernstein EE Baek JH Nierenberg AA Deckersbach T Nutrition, Exercise, and Wellness Treatment in bipolar disorder: proof of concept for a consolidated intervention. Int J Bipolar Disord (2013) 1(1):24. 10.1186/2194-7511-1-24 24660139 PMC3961757

[66] Morrato EH Newcomer JW Kamat S Baser O Harnett J Cuffel B Metabolic screening after the American Diabetes Association’s consensus statement on antipsychotic drugs and diabetes. Diabetes Care (2009) 32(6):1037–42. 10.2337/dc08-1720 PMC2681020 19244091

[67] Rathmann W Scheidt-Nave C Roden M Herder C Type 2 diabetes: prevalence and relevance of genetic and acquired factors for its prediction. Dtsch Arztebl Int (2013) 110(19):331–7. 10.3238/arztebl.2013.0331 PMC3673039 23762204

[68] Meisinger C Strassburger K Heier M Thorand B Baumeister SE Giani G Prevalence of undiagnosed diabetes and impaired glucose regulation in 35-59-year-old individuals in Southern Germany: the KORA F4 Study. Diabetes Med (2010) 27(3):360–2. 10.1111/j.1464-5491.2009.02905.x 20536501

[69] Beagley J Guariguata L Weil C Motala AA Global estimates of undiagnosed diabetes in adults. Diabetes Res Clin Pract (2014) 103(2):150–60. 10.1016/j.diabres.2013.11.001 24300018

[70] Vancampfort D Correll CU Galling B Probst M De Hert M Ward PB Diabetes mellitus in people with schizophrenia, bipolar disorder and major depressive disorder: a systematic review and large scale meta-analysis. World Psychiatry (2016) 15(2):166–74. 10.1002/wps.20309 PMC4911762 27265707

[71] Volzke H Ittermann T Schmidt CO Baumeister SE Schipf S Alte D Prevalence trends in lifestyle-related risk factors. Dtsch Arztebl Int (2015) 112(11):185–92. 10.3238/arztebl.2015.0185 PMC4390827 25837860

